# How and for whom does web-based acceptance and commitment therapy work? Mediation and moderation analyses of web-based ACT for depressive symptoms

**DOI:** 10.1186/s12888-016-0841-6

**Published:** 2016-05-23

**Authors:** Wendy T. M. Pots, Hester R. Trompetter, Karlein M. G. Schreurs, Ernst T. Bohlmeijer

**Affiliations:** Department of Psychology, Health & Technology, Centre for eHealth and Well-being Research, University of Twente, Cubicus, Drienerlolaan 5, PO Box 217, 7500 AE Enschede, The Netherlands; Dimence, Community Mental Health Centre, Haven Noordzijde 45, 7607 ES Almelo, The Netherlands; Roessingh, Research and Development, Roessinghsbleekweg 33b, 7522 AH Enschede, The Netherlands

**Keywords:** Acceptance and commitment therapy, Mindfulness, Depression, Public mental health, Randomized controlled trial, Mediation, Moderation, Prediction

## Abstract

**Background:**

Acceptance and Commitment Therapy (ACT) has been demonstrated to be effective in reducing depressive symptoms. However, little is known how and for whom therapeutic change occurs, specifically in web-based interventions. This study focuses on the mediators, moderators and predictors of change during a web-based ACT intervention.

**Methods:**

Data from 236 adults from the general population with mild to moderate depressive symptoms, randomized to either web-based ACT (*n* = 82) or one of two control conditions (web-based Expressive Writing (EW; *n* = 67) and a waiting list (*n* = 87)), were analysed. Single and multiple mediation analyses, and exploratory linear regression analyses were performed using PROCESS and linear regression analyses, to examine mediators, moderators and predictors on pre- to post- and follow-up treatment change of depressive symptoms.

**Results:**

The treatment effect of ACT versus the waiting list was mediated by psychological flexibility and two mindfulness facets. The treatment effect of ACT versus EW was not significantly mediated. The moderator analyses demonstrated that the effects of web-based ACT did not vary according to baseline patient characteristics when compared to both control groups. However, higher baseline depressive symptoms and positive mental health and lower baseline anxiety were identified as predictors of outcome across all conditions. Similar results are found for follow-up.

**Conclusions:**

The findings of this study corroborate the evidence that psychological flexibility and mindfulness are distinct process mechanisms that mediate the effects of web-based ACT intervention. The results indicate that there are no restrictions to the allocation of web-based ACT intervention and that web-based ACT can work for different subpopulations.

**Trial registration:**

Netherlands Trial Register NTR2736. Registered 6 February 2011.

## Background

Acceptance and Commitment Therapy (ACT) is an empirically based behavioural cognitive therapy that uses acceptance and mindfulness strategies together with commitment and behaviour change strategies, to increase the ability to act in accordance with personal values in the presence of life adversities [1, 2]. The effectiveness of ACT in reducing depressive symptoms has been established in several recent meta-analyses [3–5]. In addition, there is growing evidence for the efficacy of *web-based* ACT interventions [6–11]. Besides evaluating the efficacy and effectiveness of psychological treatments, randomized controlled trials can be valuable in revealing how (i.e., mediators) and for whom (i.e., moderators) therapeutic change occurs. Mediators and moderators are important to the further optimization of treatments and their clinical and cost-effectiveness. Specifically, knowledge of mediators of change enables treatment components to be included that are crucial to recovery [12]. Furthermore, both nonspecific treatment predictors and specific treatment moderators indicate who benefits from treatment, or under which conditions treatment works best [13]. Moderators and predictors can help tailor interventions to different subpopulations with possibly different causal mechanisms of disorders and ultimately improve clinical decision about treatment [13, 14]. Research into potential mediators and moderators of action in web-based ACT interventions is however in its infancy. The focus of this study is therefore on the possible mediators, moderators and predictors of change during a web-based ACT intervention.

The central therapeutic mechanism in ACT is psychological flexibility, which is the ability to act in accordance with intrinsically motivating values or goals while being in contact with the present moment [1, 2]. People who are psychological flexible also score high on acceptance, which is seen as a more effective strategy for regulating negative emotions and thoughts than experiential avoidance, i.e., the persistent and generally fruitless attempts to avoid unwanted private experiences such as feelings, thoughts, and bodily sensations [1, 15, 16]. In the model underlying ACT psychological flexibility is defined by six interrelated therapeutic processes: acceptance, cognitive defusion, contact with the present moment, self-as-context, values, and committed action. Mindfulness is taught in the context of the first four processes of the ACT model, where exercises are used to enhance an observing and non-judging self, together with the awareness and acceptance of unwanted private experiences such as thoughts and feelings. Mindfulness is often referred to as intentionally paying attention to present moment experiences in a non-judgemental way [17]. Mindfulness, as measured by the Five Facet Mindfulness Questionnaire (FFMQ), is conceptualized in several facets, which are observing (noticing or attending to internal and external experiences), describing (labelling internal experiences with words), acting with awareness (attending to one’s activities of the moment), non-judging of inner experience (taking a non-evaluative stance toward thoughts and feelings), and non-reactivity to inner experience (allowing thoughts and feelings to come and go). Baer et al. [18] stated that non-reactivity and non-judging may be seen as ways of operationalizing acceptance. They found a correlation of *r* = .49 between the Acceptance and Action Questionnaire-II (AAQ-II; measuring psychological flexibility) and the non-judging facet of the FFMQ. Although the AAQ-II and FFMQ are not meant to measure the same construct, Fledderus et al. [19] found that the AAQ-II was positively related to mindfulness facets and positive mental health and negatively related to depression and anxiety. The mindfulness facets correlated significantly with the AAQ-II, with – in agreement with Bear [18] - the strongest relation between AAQ-II and non-judging. The strongest relation between AAQ-II and non-judging (*r* = .54) suggest that the AAQ-II and the non-judging facet of the FFMQ measure are related but distinct constructs. The results also showed that the AAQ-II explains additional variance in relevant outcomes such as depression, anxiety, and positive mental health. To date, there are several studies that have suggested that augmented mindfulness mediates the effects of an ACT intervention (e.g., [20–22]). Research into psychological flexibility as a mediator in ACT interventions confirms that psychological flexibility is a core component of the theoretical framework of ACT (e.g., [1, 15, 20, 23, 24]).

As opposed to face-to-face, web-based interventions offer advantages in availability and accessibility, but non-adherence or high drop-out is an issue [25, 26]. Some researchers have suggested that in web-based interventions specificity of effects can account for the higher drop-out rates that are common in web-based interventions [14, 25]. Given this higher specificity of effects and possible individual variability in self-support via web-based interventions, it is important to study moderators and predictors. Yet, little is known about the moderators of change of *web-based* ACT interventions, and research into moderator and predictor analyses of web-based ACT interventions for depressions is lacking. There is some limited research on moderators and predictors in face-to-face ACT interventions for anxiety, indicating that factors such as socio-demographic characteristics do not moderate or predict outcome, and that factors such as mood disorder comorbidity may be predictors and/or moderators [24, 27, 28]. Also, a study by Flaxman and Bond [29] on worksite stress management training (SMT), based on ACT, showed that the impact of SMT was significantly moderated by baseline distress. For mediators of change, the number of studies doing formal mediational analyses of psychological flexibility and mindfulness in web-based ACT interventions is small [7, 30]. In the study of Trompetter et al. [30] improvements in psychological flexibility mediated the effect of web-based ACT on psychological distress, further substantiating that psychological flexibility is a core component of the ACT model. Also, Bricker et al. [7] found that acceptance processes mediated the effects on smoking cessation through greater acceptance of physical urges, cognitions, and emotions.

The aim of the current study was to identify mediators, moderators and predictors of participant improvement in the web-based ACT intervention in a recently published randomized controlled trial (RCT) [11]. The primary objective was to test the hypothesis that pre- to post-treatment changes in psychological flexibility and mindfulness mediated the effects of the web-based ACT intervention on depressive symptoms. A second objective was to explore which participant baseline characteristics and baseline symptoms moderated or predicted treatment effects. These latter analyses were considered exploratory given the lack of research so far.

## Methods

### Participants and procedure

The sample for the current study stems from the original sample in the RCT on the effectiveness of web-based public mental health intervention based on ACT [11]. The study was approved by an independent medical ethics committee for research in mental health settings in the Netherlands (METiGG; number NL33619.097.100). In addition, this study has been recorded in the Dutch primary trial register for clinical trials (Netherlands Trial Register, NTR2736). In brief, participants with mild to moderate depressive symptoms were recruited through advertisements in Dutch national newspapers and on the Internet. A webpage created for the purpose of this study included an outline of the study design, and a registration possibility for participation in the trial. Within the webpage candidates filled out a secured computerized informed consent. After receiving the informed consent, initial screening was conducted online for checking the in- and exclusion criteria by use of a self-report questionnaire in a fully automated computerized assessment battery. Study inclusion criteria were: a) an age of 18 years or older with mild to moderate depressive symptoms (> 10 on the Dutch version of the Center of Epidemiological Studies Depression Scale; CES-D [31]), and b) completion of the baseline measurement. Applicants were excluded if on initial screening they reported: (a) few depressive symptoms (≤10 on the CES-D); (b) received psychological or psychopharmacological treatment for mental complaints within the last three months; (c) reading or writing problems due to insufficient Dutch language skills; and (d) an inability to invest approximately 30 min per day up to three hours per week in the intervention and daily practices. Furthermore, participants with severe psychopathology were excluded when diagnosed with a current severe mental disorder or when having a moderate to high suicide risk, according to the Dutch version of the Mini International Neuropsychiatric Interview [32, 33] and the Sheehan Disability Scale [34].

**Fig. 1:**
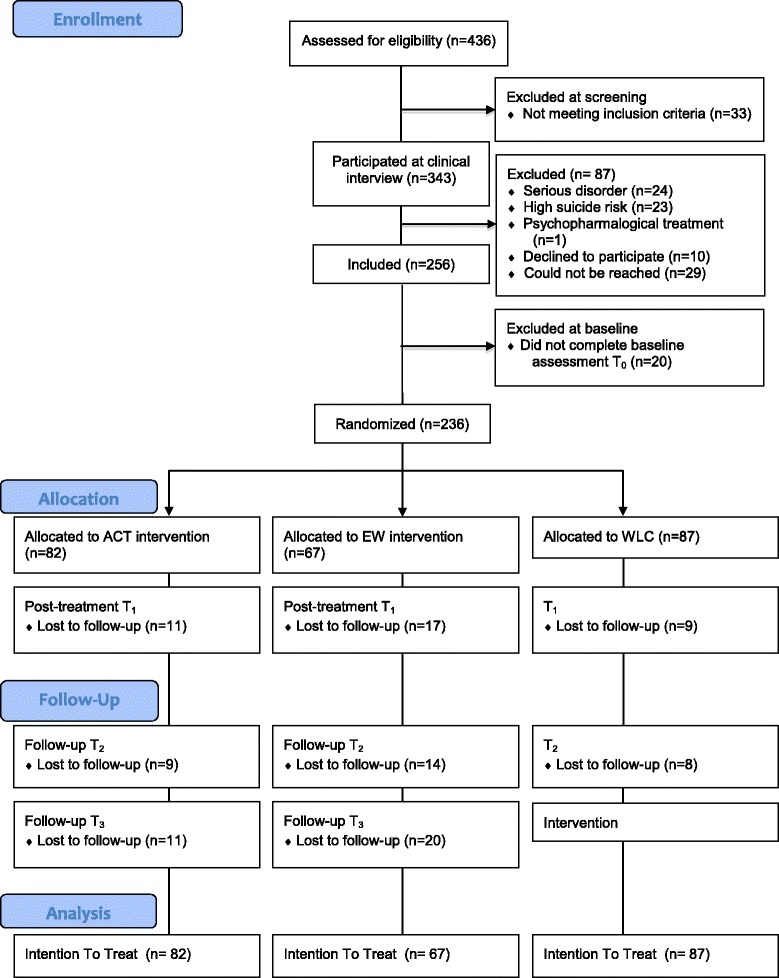
CONSORT flow of the participants

Of the 436 persons that were assessed for eligibility, 236 participants were randomly allocated to either ACT (*n* = 82), an active control condition based on Expressive writing (EW; *n* = 67) or to a waiting list control condition (WLC; *n* = 87). Figure 1 shows the CONSORT flow of the participants. Baseline characteristics can be found in Table 1. Due to a programming error in the randomization procedure, the number of participants in each condition differed. There were no significant differences at baseline between the conditions for any of the demographic variables or outcome measures, except for gender [*X*^*2*^ (2, *n* = 236) = 22.78, *p* < 0.00; percentage of female was higher in the ACT group, followed by WLC, followed by EW].

**Table 1:** Baseline characteristics

Characteristic	Total (*n* = 236)	ACT (*n* = 82)	EW (*n* = 67)	WLC (*n* = 87)
Age, mean, y (SD)	46.85 (12.06)	45.15 (10.78)	46.73 (12.65)	48.54 (12.63)
Range	20–73	21–69	20–69	21–73
Gender, No. (%)				
Female	179 (75.8)	76 (92.7)	40 (59.7)	63 (72.4)
Male	57 (24.2)	6 (7.3)	27 (40.3)	24 (27.6)
Education, No. (%)				
High	157 (66.5)	55 (67.1)	45 (67.2)	57 (65.5)
Middle	75 (31.8)	24 (29.3)	22 (32.8)	29 (33.3)
Low	4 (1.7)	3 (3.7)	0 (0.0)	1 (1.1)
Diagnosis, No. (%)				
No diagnosis	97 (41.1)	36 (43.9)	22 (32.8)	39 (44.8)
Major Depressive episode	18 (7.6)	7 (8.6)	6 (9.0)	5 (5.8)
Recurrent depression	61 (25.9)	21 (25.6)	21 (31.3)	19 (21.8)
Dysthymic disorder	5 (2.1)	0 (0.0)	3 (4.5)	2 (2.3)
Other mood disorders	11 (4.7)	2 (2.4)	4 (6.0)	5 (5.8)
Anxiety disorder	44 (18.6)	16 (19.5)	11 (16.4)	17 (19.5)
Comorbidity, No. (%)				
Mood disorder	54 (22.9)	22 (26.8)	17 (25.4)	15 (17.2)
Anxiety disorders	3 (1.3)	0 (0.0)	3 (4.5)	0 (0.0)

*ACT* Acceptance and Commitment Therapy, *EW* Expressive Writing, *WLC* Waiting List Condition

### Intervention

In the current study an ACT-protocol called ‘Living to the full’ [35] was used, which has an explicit focus on mindfulness [11, 35]. The web-based ACT intervention comprised of nine online modules, that could be worked through in nine to twelve weeks. This self-help intervention has shown to be effective in reducing depressive and anxiety symptoms, and improving positive mental health, psychological flexibility and mindfulness, as a group course and as a bibliotherapy intervention with e-mail support [31, 36, 37]. The nine sessions are based on six core processes of ACT that together promote psychological flexibility [2]. Each module uses experiential exercises and metaphors to illustrate the ACT processes, text messages, tailored stories for motivation, and an option to personalize the homepage. Furthermore, participants were encouraged to practice daily mindfulness exercises that were provided on audio, downloadable within the web-based intervention. Weekly e-mail counselling by trained graduate psychology students was offered for personal feedback, encouragement and support. For a comprehensive description of the development of the intervention see Kelders et al. [38].

The active control condition was a web-based Expressive Writing (EW) intervention based on Pennebaker’s expressive writing paradigm [39], and comprised of nine online sessions that could be worked through in nine to twelve weeks. Every session started with psycho-education on emotions and emotion regulation, and was followed by instructions of the method of EW. In general, people were instructed to write about emotional experiences for 15 to 30 min on at least three days during one week. E-mail counselling was offered similarly to the web-based ACT intervention.

Participants in the waiting list condition were offered no intervention and were free to access other forms of care. Six months after baseline these participants could start a web-based intervention of choice.

### Summary of previously reported results from the RCT

As reported in Pots et al. [11], analyses using repeated measures ANOVA showed that the web-based ACT intervention was superior to both the active control condition (EW intervention) and the WLC, on all outcome measures (depressive symptoms, anxiety and positive mental health) and process variables (psychological flexibility and mindfulness, except for the mindfulness facets *observing* and *acting with awareness*). The effect sizes at post-treatment were small to moderate and ranged from 0.35 to 0.56. The effects of the web-based ACT intervention were maintained at 6- and 12-month follow up. However, at follow-up there were no significant differences between the web-based ACT intervention and the web-based EW intervention or the WLC. Also, as there was no restricted access to care for the WLC, non-study treatment was checked but revealed no difference between the conditions. Overall, the results showed that on the short term the web-based ACT intervention was significantly more effective to both control conditions, but that both web-based interventions had similar effects on depressive symptoms and secondary measures at 6- and 12-month follow-up.

### Measures

In this study, we used the outcome measures of the RCT assessed at baseline, at post-treatment 3 months after baseline (directly after the intervention), and at 6- and 12-month follow-up. The process variables were used as possible mediators. All other measures functioned as possible moderators/predictors of change and were assessed at baseline, prior to randomization.

As outcome measures the Dutch version of the Center of Epidemiological Studies - Depression Scale was used. The CES-D (20 items, score 0–60) measures symptoms of depression in the general population. Respondents rate to what extent they experienced depressive symptoms in the previous week. Higher scores mean more depressive symptoms [40, 41], and a score of 16 or higher is indicative of clinically relevant depressive symptoms [42, 43].

Measures for mediation were the Acceptance and Action Questionnaire-II (AAQ-II), measuring psychological flexibility, and the Five Facet Mindfulness Questionnaire – Short Form (FFMQ-SF), measuring mindfulness. The AAQ-II (10 items, score 10–70) measures the subject’s willingness to be in contact with negative private events, the acceptance of these events, and the ability to live according to his/her values. Higher scores indicate more psychological flexibility [19, 44]. The FFMQ-SF (24 items, score 24–120) was used to measure mindfulness in five sub-dimensions: (1) observing (4 items), defined in terms of noticing or attending to internal and external experiences; (2) describing (5 items), defined in terms of labelling internal experiences with words; (3) acting with awareness (5 items), defined in terms of attending to one’s activities of the moment; (4) non-judging of inner experience (5 items), defined in terms of taking a non-evaluative stance toward thoughts and feelings; and (5) non-reactivity to inner experience (5 items), defined in terms of allowing thoughts and feelings to come and go, without getting caught up in or carried away by them. Facet scores range from 4 to 25 (except for *observing*, which ranges from 4 to 20), with higher scores indicating more mindfulness [18, 45].

Measures for moderation and prediction were demographic variables, depressive symptoms, anxiety symptoms, positive mental health, and diagnostic classification. Demographic variables that were assessed as possible moderators/predictors were age, gender, and educational level. For depressive symptoms the baseline score of the CES-D was also used as a moderator/predictor. For anxiety symptoms the Hospital Anxiety and Depression Scale - Anxiety subscale (HADS-A; 7 items, score 0–21) measures symptoms of anxiety. Higher scores mean more anxiety symptoms [46, 47]. Positive mental health was measured with the Mental Health Continuum- Short Form (MHC-SF; 14 items, score 0–5) measures three dimensions of positive mental health: (1) emotional well-being, defined as the presence of positive feelings/satisfaction with life; (2) social well-being, defined as positive functioning in community life; and (3) psychological well-being, defined as positive functioning in individual life. In this study the total MHC-SF score was used, with higher scores indicating greater emotional, social and psychological well-being [48, 49]. Lastly, diagnostic classification was measured with the Dutch version of the Mini-International Neuropsychiatric Interview (MINI) [32, 33], supplemented with the Sheehan Disability Scale (SDS) [34], was used to measure the severity of a disorder. Severity was defined as at least two areas of role functioning with severe role impairment due to the disorder according to the SDS. The MINI and SDS were conducted by telephone at baseline. Diagnostic classifications that were assessed as possible moderators/predictors were current disorder, current depressive disorder, and comorbidity.

### Analyses

Statistical analyses were in agreement with the Intention-to-treat (ITT) principle. ITT analyses were performed using the SPSS Missing Value Analysis to impute all missing data on the continuous measures with the expectation-maximization (EM) method [50]. Available data was 96.6 % at baseline and 84.5 % at post-treatment. Prior to the main analyses, one-way analysis of variance (ANOVA) and chi-square tests were conducted to examine baseline differences in all potential moderators/predictor variables between the web-based ACT intervention and the two control conditions. Overall, significance of mediators, moderators and predictors were interpreted at *p* < 0.05. The statistical analyses were performed using SPSS 20.

Both simple and multiple mediation analyses were performed using PROCESS [51]. PROCESS is based on regression-based path-analytic framework and combines mediation and moderation into one conditional process model. PROCESS estimates the indirect effect and bias-corrected confidence intervals (CI). All analyses were based on 5000 bootstrapping samples. An indirect effect was considered significant when the CI did not include zero. The change score (baseline to post-treatment, baseline to 6-months, baseline to 12-months) on the outcome measure CES-D was entered as the dependent variable. The dummy variable representing treatment (ACT = 1, WLC = 0 or EW = 0) and the potential mediator (measured as the change score baseline to post-treatment, baseline to 6 months, baseline to 12 months) were entered as independent variables. To control for variation in outcome score the baseline score of CES-D was entered as a covariate. Analyses were done separately for the web-based ACT intervention compared to WLC, and the web-based ACT intervention compared to the web-based EW intervention.

Linear regression models were applied for moderation variables using the PROCESS macro for SPSS. During the analyses, each potential moderator was mean centered. The change score (baseline to post-treatment, baseline to 6 months, baseline to 12 months) on the outcome measure CES-D was entered as the dependent variable. The dummy variable representing treatment, the mean centred potential moderator, and the treatment by mean centered moderator variable were entered as independent variables. To control for variation in outcome score, also in the moderator analyses, the baseline score of CES-D was entered as a covariate. Analyses were done separately for the web-based ACT intervention compared to WLC, and compared to the web-based EW intervention. In general, when there was a significant interaction effect the variable entered was interpreted as being a moderator of change.

For the predictor analyses linear regression analyses across all three groups were performed using SPSS 22, with the change score of depressive symptoms (baseline to post-treatment, baseline to 6 months, baseline to 12 months) as the dependent variable and the presumed predictors as independent variables. The outcome was adjusted for baseline values of depression by adding the baseline score of the CES-D as an additional independent variable in the regression. Also, multiple linear regressions were performed by simultaneously entering all proposed predictors as independent variables into the multiple regression. On account of the exploratory nature of the moderator and predictor analyses the borderline *p*-value was not adjusted.

## Results

### Mediation analyses

**Fig. 2:**
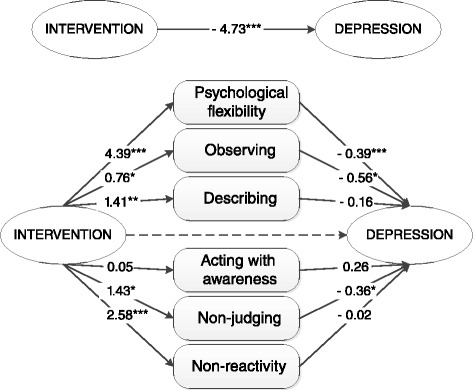
Multiple mediation of psychological flexibility and mindfulness as mediators of the ACT intervention compared to the WLC. Describe, describing; Observe, observing; Act aware, acting with awareness; Non-judging, non-judging of inner experience; Nonreactivity, non-reactivity to inner experience. *P < .05; **P < .01; ***P < .001

Table 2 shows the results of the outcomes of the simple mediation analyses. The web-based ACT intervention, as compared to the WLC, showed significantly greater improvement on the primary outcome measure post-treatment (total treatment effect β = −4.73, *p* < .001). The bootstrap results for the indirect effects of the web-based ACT intervention on the WLC showed that changes in psychological flexibility and all mindfulness facets (except *acting with awareness*) significantly mediated the effect of the web-based ACT intervention on depressive symptoms post-treatment. Figure 2 shows the results of the multiple mediation model, comparing the web-based ACT intervention to the WLC. When all mediators were entered into the model, three process variables remained significant mediators of the effect on depressive symptoms: psychological flexibility (indirect effect β = −1.691, 95 % CI −2.96 to −0.76), and the mindfulness facets *observing* (indirect effect β = −0.428, 95 % CI −1.17 to −0.02) and *non-judging of inner experience* (indirect effect β = −0.518, 95 % CI −1.52 to −0.10). Follow-up analyses from T_0_ to T_2_ revealed somewhat different outcomes, with no significant mediating effect for the mindfulness facet *observing* (indirect effect β = −0.2854, 95 % CI −1.12 to 0.40). Psychological flexibility (indirect effect β = −1.9441, 95 % CI −3.52 to −0.77), and the mindfulness facets *describing* (indirect effect β = −1.0622, 95 % CI −2.35 to −0.17), *non-judging of inner experience* (indirect effect β = −1.7709, 95 % CI −3.32 to −0.82) and *non-reactivity to inner experience* (indirect effect β = −1.7766, 95 % CI −3.30 to −0.74) remained significant mediators. When all mediators were entered into the multiple mediation model results on the follow-up analyses showed three remaining process variables: psychological flexibility (indirect effect β = −1.5605, 95 % CI −3.17 to −0.56) and the mindfulness facets *describing* (indirect effect β = −0.4896, 95 % CI −1.63 to −0.00) and *non-judging of inner experience* (indirect effect β = −0.9862, 95 % CI −2.23 to −0.23).

**Table 2:** Outcomes of simple mediation analyses from baseline to post-treatment assessing indirect effects of the ACT intervention through process variables on the outcome measure CES-D compared to both control conditions^a^

				Bootstrap results for indirect effects (95 % CI)		
	c’-path	a-path	b-path	ab	Lower	Upper
ACT vs WLC						
Psychological flexibility	−2.81*	4.39***	−0.44***	−1.9220*	−3.22	−1.01
FFMQ - observing	−4.18***	0.76*	−0.73**	−0.5584*	−1.37	−0.09
FFMQ - describing	−4.16***	1.41**	−0.41	−0.5740*	−1.56	−0.04
FFMQ - acting with awareness	−4.73***	0.05	−0.14	−0.0064	−0.34	0.16
FFMQ - non-judging of inner experience	−3.89**	1.43*	−0.59***	−0.8483*	−2.06	−0.04
FFMQ - non-reactivity to inner experience	−3.41**	2.58***	−0.51**	−1.3224*	−2.54	−0.53
ACT vs EW						
Psychological flexibility	−2.02	1.89	−0.36***	−0.6816	−1.50	0.09
FFMQ - observing	−2.72*	−0.08	−0.24	0.0184	−0.14	0.40
FFMQ - describing	−2.52*	0.52	−0.36	−0.1872	−0.89	0.07
FFMQ - acting with awareness	−2.70*	−0.53	0.00	−0.0005	−0.00	0.33
FFMQ - non-judging of inner experience	−1.92	0.94	−0.83***	−0.7794	−1.91	0.02
FFMQ - non-reactivity to inner experience	−1.72	1.37**	−0.72***	−0.9876*	−2.12	−0.31

*ACT* Acceptance and Commitment Therapy, *WLC* Waiting List Condition, *EW* Expressive Writing, *CES-D* Center for Epidemiological Studies – Depression Scale, *FFMQ* Five Facet Mindfulness Questionnaire

^a^Values are unstandardized Betas;* Significant at *p* < .05; ** Significant at *p* < .01; *** Significant at *p* < .001

The total treatment effect of the web-based ACT intervention compared to the web-based EW intervention showed a significantly greater improvement on the primary outcome measure post-treatment (β = −2.70, *p* = 0.030). When the indirect effects of the web-based ACT intervention were compared with the web-based EW intervention, only change in the mindfulness facet *non-reactivity to inner experience* appeared as mediator of the effect on depressive symptoms. When all mediators were entered in the multiple mediation model no significant mediators remained. In the follow-up analyses the mindfulness facet *non-reactivity to inner experience* remained a significant mediator in the simple mediation model (T_0_ to T_2_, indirect effect β = −0.9792, 95 % CI −2.24 to −0.20; T_0_ to T_3_, indirect effect β = −0.8149, 95 % CI −1.92 to −0.09). When all mediators were entered in the multiple mediation model, only from T_0_ to T_1_ the mindfulness facet *non-reactivity to inner experience* remained a significant mediator (indirect effect β = −0.5194, 95 % -1.54 to −0.00). No significant mediators remained for T_0_ to T_2_ and T_0_ to T_3_.

### Moderation and predictor analyses

Table 3 shows the results of the moderator analyses for the web-based ACT intervention compared to the WLC and the web-based EW intervention respectively from baseline to post-treatment. Compared to both control conditions no single significant moderator emerged, indicating that there were no significant differences for demographic characteristics, psychological measures, diagnostic classification or comorbidity. Follow-up analyses showed similar results, except for current disorder at T_0_ when comparing the web-based ACT intervention versus the web-based EW intervention from baseline to 12 months (T_0_ to T_3_). Participants in the web-based ACT intervention having a current disorder at T_0_ had more change in depressive symptoms from baseline to 12 months, when compared to the web-based EW intervention. No other significant moderators were found from T_0_ to T_2_ or from T_0_ to T_3_, for both ACT versus WLC and ACT versus EW.

**Table 3:** Results of moderator analyses of the ACT intervention on the outcome measure CES-D post-treatment compared to both control conditions^a^

	ACT vs WLC				ACT vs EW			
		95 % CI				95 % CI		
	b	lower	upper	*p*	b	lower	upper	*p*
Demographic characteristics								
Age	−0.101	−0.32	0.12	.366	−0.026	−0.24	0.19	.810
Gender	−1.253	−10.29	7.78	.785	−0.375	−9.20	8.45	.933
Educational level	1.190	−0.70	3.08	.215	0.429	−1.55	2.40	.668
Psychological measures								
Depression (CES-D)	−0.228	−0.53	0.07	.133	−0.118	−0.39	0.15	.393
Anxiety (HADS-A)	−0.675	−1.40	0.05	.068	−0.267	−1.03	0.50	.492
Emotional well-being (MHC)	0.223	−2.08	2.53	.849	−1.014	−3.71	1.68	.459
Psychological well-being (MHC)	0.556	−1.91	3.02	.656	−0.589	−3.21	2.04	.658
Social well-being (MHC)	0.325	−1.99	2.64	.782	0.767	−1.69	3.23	.539
Diagnostic classification								
Current disorder	1.640	−3.47	6.75	.527	−1.332	−6.54	3.88	.614
Current depressive disorder	1.294	−3.99	6.58	.629	0.767	−4.39	5.93	.769
Comorbidity	0.775	−5.49	7.04	.807	−1.195	−7.08	4.69	.689

*ACT* Acceptance and Commitment Therapy, *WLC* Waiting List Condition, *EW* Expressive Writing, *CES-D* Center for Epidemiological Studies – Depression Scale, *HADS-A* Hamilton Anxiety and Depression Scale – Anxiety subscale, *MHC* Mental Health Continuum

^a^Values are unstandardized Betas

Table 4 shows the variables that were statistically significant predictors of change in depressive symptoms over time across all conditions. Participants reporting more depressive symptoms and positive mental health (emotional, social and psychological well-being) at baseline had greater change in depressive symptoms from pre- to post-treatment, adjusted for baseline depressive symptoms. However, participants with more anxiety symptoms had less change in depressive symptoms post-treatment, adjusted for baseline depressive symptoms. No predictor effects were found for age, gender, level of education, or diagnostic classification. When all proposed predictors were entered into the multivariate regression model only baseline depressive and anxiety symptoms and emotional well-being remained significant predictors. Follow-up results on the predictor analyses showed similar results on the univariate analyses from T_0_ to T_2_. Follow-up results from T_0_ to T_3_ showed lack of significant predictor effect for baseline anxiety, supplemented with an additional significant predictor of level of education from pre- to 12 months. This indicates that participants reporting a higher level of education at baseline, more depressive symptoms and positive mental health (emotional, social and psychological well-being) at baseline experienced a greater change in depressive symptoms from pre- to 12 months, adjusted for baseline depressive symptoms. When all proposed predictors were entered into the multivariate model somewhat different results appeared. At 6 months follow-up baseline depressive and anxiety symptoms and psychological well-being remained significant predictors of change in depressive symptoms, and at 12 months follow-up only baseline depressive symptoms and psychological well-being remained a significant predictor of change in depressive symptoms.

**Table 4:** Results of predictor analyses on the outcome measure CES-D over time collapsing across all conditions^a^

	Univariate			Multivariate		
	T0-T1	T0-T2	T0-T3	T0-T1	T0-T2	T0-T3
Demographic characteristics						
Age	0.004	0.085	0.014	0.017	0.059	−0.012
Gender	−1.806	−2.407	−1.394	−1.314	−1.689	−0.562
Educational level	−0.627	−0.781	−1.230*	−0.330	−0.498	−0.908
Psychological measures						
Depression (CES-D)	−0.619***	−0.628***	−0.594***	−0.835***	−0.816***	0.196***
Anxiety (HADS-A)	0.825***	0.784**	0.398	0.662***	0.733**	0.374
Emotional well-being (MHC)	−2.993***	−2.833***	−2.626**	−1.711*	−1.018	−0.590
Psychological well-being (MHC)	−3.153***	−3.549***	−4.089***	−1.406	−2.074*	−3.356**
Social well-being (MHC)	−2.109***	−2.170**	−2.582**	−0.514	−0.628	−0.359
Diagnostic classification						
Current disorder	0.415	−0.678	0.271	−0.093	−0.620	−0.122
Current depressive disorder	0.004	−0.684	1.201	−1.578	−1.158	1.264
Comorbidity	1.447	−0.755	−0.668	0.451	−1.628	−2.160

*CES-D* Center for Epidemiological Studies – Depression Scale, *HADS-A* Hamilton Anxiety and Depression Scale –Anxiety subscale, *MHC* Mental Health Continuum

^a^Outcome adjusted for baseline value of depression; Values are unstandardized Betas;*P < .05, **P < .01, ***P < .001

## Discussion

The aim of the present study was to examine why and how a web-based ACT intervention for depressive symptoms is effective, and to identify for whom and under what circumstances this intervention has differential effects. Psychological flexibility and two mindfulness facets were the strongest mediators of the treatment effect of the ACT intervention versus the WLC. Only change in the mindfulness facet *non-reactivity to inner experience* appeared as mediator of the treatment effect of the ACT intervention versus the web-based EW intervention. The moderator analyses demonstrated that the effects of the web-based ACT intervention on depressive symptoms did not vary according to baseline patient characteristics when compared to both control groups. However, baseline symptoms and positive mental health were identified as predictors of outcome when collapsing across all conditions. Follow-up results on mediation and moderation/prediction were somewhat similar.

The findings of this study on mediation corroborate the evidence that psychological flexibility and mindfulness are distinct process mechanisms that mediate the effect of web-based ACT intervention on depressive symptoms. This confirms the relevance of psychological flexibility as the central therapeutic mechanism in the ACT model and further strengthens the theoretical framework of ACT [2]. Furthermore, our results give empirical support for mindfulness as an important mediator in the ACT model. Both the processes of psychological flexibility and mindfulness can be considered within a broader context of emotion regulation as a transdiagnostic mechanism of change in mindfulness- and acceptance based interventions (e.g., [52–54]). Chiesa et al. [55] suggest that such interventions may in general enhance positive emotional regulation strategies, as well as self-compassion levels, and decrease rumination and experiential avoidance. They specifically suggested that short-term mindfulness meditation practitioners might achieve these benefits by means of a top-down regulation of the prefrontal areas on limbic areas [52]. Hence, the studies on the broader context of emotion regulation endorse mindfulness and psychological flexibility processes as an emotion regulation process within the theoretical framework of ACT.

Mediation analyses of ACT versus EW showed that no mediators remained significant in the follow-up analyses of the multiple mediation, in contrast to the simple mediation. In the previously reported results from the RCT [11], we found that the web-based ACT intervention had similar effects on depressive symptoms and secondary measures after 6 and 12 months compared to the EW intervention. Additionally, the web-based EW condition showed significant improvement compared with the WLC from baseline to follow-up (T_0_ to T_2_) for psychological flexibility and some facets of mindfulness, and no significant improvement on other outcome measures. The results of the current study show that there is no unique mediator of change when comparing the web-based ACT intervention versus the web-based EW intervention. These findings, combined with the results from the RCT, suggest that the web-based EW intervention shares important therapeutic mechanisms with the web-based ACT intervention, notably psychological flexibility and facets of mindfulness. This lack of specificity is not an isolated finding. A recent meta-analysis of Goyal et al. [56] found that studies comparing a mindfulness intervention with comparison conditions (active controls) showed no significant differences on the FFMQ or other mindfulness measures. Goldberg et al. [57] compared a Mindfulness-based Stress Reduction program with an active control condition that did not include the instruction in mindfulness meditation, and a waiting list control. In accordance with our results their active control condition also showed an improvement over time on the FFMQ, suggesting that their active control condition did induce mindfulness although it was not specifically trained. The literature on the processes of change in EW is however still in its infancy. Some researchers have indicated that acceptance or experiential disclosure is an important mechanism [39, 58]. The results of this study confirm the concept that acceptance is a process mechanism of EW as both psychological flexibility and mindfulness encompass acceptance, but future studies are necessary to shed more light on the therapeutic mechanisms in EW. This might, however, prove difficult in lack of a solid theoretical model underlying EW.

A second aim of this study was to explore moderators and predictors of change to identify characteristics of those likely to benefit. Since none of the potential moderators were significant for ACT versus WLC and these effects were maintained in the follow-up analyses, the results indicate that there are no restrictions to the allocation of the web-based ACT intervention and that the web-based ACT intervention can work for different subpopulations. This is a significant finding, as web-based interventions can have a large incremental impact in availability and accessibility of interventions [59–61]. To attain this impact, however, it is highly necessary for web-based interventions to ensure treatment adherence in addition to treatment effectiveness. The high adherence in this study might indicate feasibility and compatibility with the users by using a holistic approach based on persuasive technology, such as the usage of SMS support, multi-media, feedback and tailoring, which has been shown to be positively related to adherence [62]. This might indicate that the persuasive technology used in the web-based ACT intervention made the intervention more suitable to individual’s needs. When comparing the web-based ACT intervention versus the web-based EW intervention similar results were found, except for a single moderator at 12 months follow-up, notably having a current disorder at baseline. This result is interesting as it indicates that having a current disorder at baseline is predictive of more improvement in depressive symptoms at 12 months follow-up, when receiving ACT versus EW. No other moderators were found for ACT versus EW. In short, the results suggest that the level of symptoms is more severe ACT seems to be more beneficial at long term follow-up, compared to EW. In addition to moderator analyses the predictor analyses indicated that participants reporting more depressive symptoms, and emotional, psychological and social well-being at baseline, had greater change in depressive symptoms from pre- to post-treatment. However, having more anxiety symptoms at baseline seemed to lead to less change in depressive symptoms from pre-to post-treatment. Follow-up results showed similar results on the univariate analyses. In the multivariate model baseline depressive and anxiety symptoms, and psychological well-being remained significant predictors of change in depressive symptoms at 6 months follow-up, and only baseline depressive symptoms and psychological well-being remained a significant predictor of change in depressive symptoms at 12 months follow-up. These results indicate that people with more depressive symptoms and positive psychological functioning have the best opportunities for improvement. This latter finding is in accordance with the finding that positive mental health is a protective factor against mental illness [63, 64]. Previous studies in different populations on moderators and predictors of face-to-face ACT interventions are sparse and non-conclusive on what moderators and predictors are specific to help tailor interventions to different subpopulations [24, 27–29, 31, 65]. Some studies found that greater baseline symptoms, neuroticism, and experiential avoidance are moderators or predictors, but the results are not consistent (e.g., [24, 27, 31, 65]). A recent meta-analysis on 49 randomized controlled trails Comparing computerised Cognitive Behaviour Therapy (CCBT) to other therapies and waiting list controls, sheds some light on moderators of web-based interventions [66]. The findings indicated that the benefit of CCBT decreases as age increases. Also type of control group moderated effect sizes, with higher effect sizes for comparisons of CCBT with inactive controls. Interestingly, no further significant moderating effect were found for any of the variables (demographic characteristics, severity of symptoms, type of intervention, type of support). A highly prevalent and important potential moderator is comorbidity. In this study, comorbidity was not found to be a moderator or predictor of effects, which is of importance because the majority of web-based interventions have targeted specific disorders. Interestingly, Wolitzky et al. [27] found that ACT outperformed CBT among those with comorbid mood disorders. In contrast, Niles et al. [24] also compared CBT with ACT and found no such effect. Despite the importance of these findings on face-to-face interventions, it is to be expected that results of face-to-face ACT interventions are not comparable to web-based ACT interventions as these latter are expected to place a greater burden on self-efficacy or resilience. The fact that in the current study there were no moderators found when comparing ACT versus WLC, suggests that web-based ACT can work for different subpopulations. As the analyses were exploratory, the results should be interpreted with caution. Overall, we can conclude that the studies so far that have included moderator and predictor analyses in web-based ACT interventions are sparse and mostly exploratory, highlighting the necessity of research in this area. Future research should include more formal moderator and predictor analyses to be able to better tailor individuals to treatment.

This study has several limitations. First, the RCT was not powered for the moderator analyses. Since the analyses were exploratory and post-hoc, the absence of significant moderators could well be a result of inadequate statistical power [67]. However, our study is in accordance with the methodological requirements of exploratory moderator analyses [68]. Second, all mediation analyses were performed using baseline to follow-up measurements of both processes and outcomes, precluding an evaluation of temporal precedence. Future studies could use more sophisticated designs to address temporal precedence of the process variables, such as a cross-lagged panel design [69]. Third, the original sample in the RCT was predominantly female with a rather high level of education, recruited from the general Dutch community through newspapers. This restricts the generalizability of the results to gender and education. The fact that our sample was fairly homogeneous could also be an argument for the fact that no moderators were found [29]. Fourth, in this study we focused on the overarching processes of the theoretical model ACT, psychological flexibility and mindfulness. Nowadays, more specific measures of the individual core processes of ACT such as the Engaged Living Scale [70] measuring an engaged response style, are available.

## Conclusions

Overall, this study was the first to assess mediators, moderators and predictors of change in a web-based ACT treatment for depressive symptoms. The findings demonstrate that web-based ACT is successful in enhancing psychological flexibility and facets of mindfulness and that these changes mediate the short and long-term effects on depressive symptoms. The findings also demonstrate that at present there is no reason to exclude people from web-based ACT, though when the level of depressive symptoms is more severe web-based ACT seems to be more beneficial at long term follow-up, compared to web-based EW. Furthermore, higher levels of depressive symptoms and higher levels of psychological well-being predict better long term outcomes.
